# Cultivation to improve *in vivo* solubility of overexpressed arginine deiminases in *Escherichia coli* and the enzyme characteristics

**DOI:** 10.1186/1472-6750-14-53

**Published:** 2014-06-07

**Authors:** Ying Wang, Yue-Zhong Li

**Affiliations:** 1 State Key Laboratory of Microbial Technology, School of Life Science, Shandong University, Jinan 250100, P. R. China

**Keywords:** Arginine deiminase, Heterologous expression, *Escherichia coli*, Solubilization, Cultivation, Conversion, L-arginine, L-citrulline

## Abstract

**Background:**

Overexpression of foreign genes in *Escherichia coli* cells is an efficient means to obtain recombinant proteins. The technique is, however, often hampered by misfolding, degradation, aggregation and formation in inclusion bodies of products.

**Results:**

In this study, we reported that *in vivo* solubility of overexpressed arginine deiminases (ADI) improved by changing the cultivation conditions. ADI is enzymes that convert L-arginine to L-citrulline. After codon optimization, we synthesized the ADI gene of *Pseudomonas putida* and constructed it for overexpression in *E. coli* cells. The rADI products were mainly in inclusion body forms. We performed a series of optimization to enhance solubility of the protein. Co-expression with the GroES-GroEL chaperone team increased approximately 5-fold of the rADI activity. In addition the combination of L-arginine and D-glucose in the Luria-Bertani (LB) growth medium further increased the total activity to about 15 times. Separate L-arginine and D-glucose or the addition of other saccharides or amino acids had no such effects. The solubilization effects of the combination of L-arginine and D-glucose were further confirmed in the overexpression of another ADI from *Listeria welshimeri*. The enzymatic and conversion characteristics of the rADI products were further determined.

**Conclusions:**

Combined addition of L-arginine and D-glucose in the LB medium significantly improved *in vivo* solubility of rADI proteins. The present study suggested a new strategy to increase the solubilization of overexpressed recombinant proteins in *E. coli* cells.

## Background

*Escherichia coli* cells are an efficient factory to produce foreign proteins because of high-cell density, well-known genetics background and the simplest genetic manipulation [1,2]. However, high expressions of recombinant proteins often result in misfolding, aggregation, degradation, low activity and formation in inclusion-bodies of products, which greatly hampers associated researches and industrial and medical applications [2-6]. In order to improve the solubility of recombinant proteins, many strategies have been developed, such as co-expression with chaperones, secretion expression of recombinant proteins and protein fusion techniques [7,8]. In this study, we reported that combined addition of D-glucose and L-arginine in the Luria-Bertani (LB) medium to cultivate *E. coli* cells were efficient to enhance the solubility of recombinant arginine deiminases.

Arginine deiminase (EC3.5.3.6; ADI) hydrolyzes L-arginine to L-citrulline, releasing ammonia. Arginine deiminase, combining with ornithine transcarbamylase and carbamate kinase forms the arginine deiminase pathway [9], which is widely distributed in the microorganisms that are able to utilize arginine as an energy source [10]. By depleting extracellular supply of L-arginine, ADI is a potential anti-tumor agent for its efficient inhibition effects on the growth of arginine-auxotrophic tumor cells, especially hepatocellular carcinomas and melanomas [10-12]. ADI is also considered a useful producer of L-citrulline at industrial scale [13]. So, many ADI genes from different sources, including *Mycoplasma arginini*[11,12,14-16], *Pseudomonas plecoglossicida*[17], *Lactococcus lactis*[18], *Streptococcus sanguis*[19] and *Giardia intestinalis*[20,21] have been cloned and expressed in *E. coli* cells. However, the recombinant ADI proteins were mainly existed in inclusion body forms, which is a bottleneck in its research and development. We overexpressed arginine deiminases from different resources in the *E. coli* cells for industrial conversion L-arginine to L-citrulline. Our recombined ADI proteins named rADI, were similarly mostly in inclusion bodies. In this study, we made comprehensive optimizations to improve solubility of the rADI proteins and found that the combined addition of L-arginine and D-glucose in Luria-Bertani (LB) medium distinctly increased the *in vivo* solubility of rADI in *E. coli* cells. We studied the enzymatic and conversion characteristics of the rADI.

## Results

### Construction and expression of ADI gene in *E. coli*

**Figure 1:**
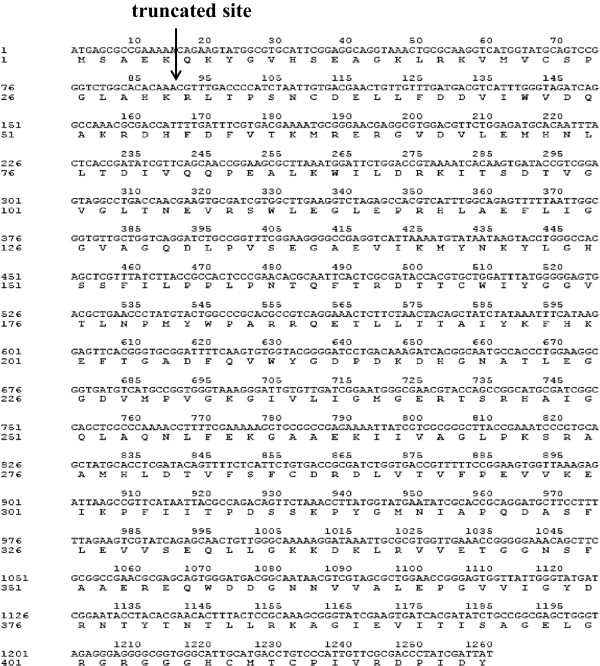
**Nucleotide and corresponding amino acid sequences of the artificial ADI gene.** The black arrow indicates the truncation site of T-rADI.

**Figure 2:**
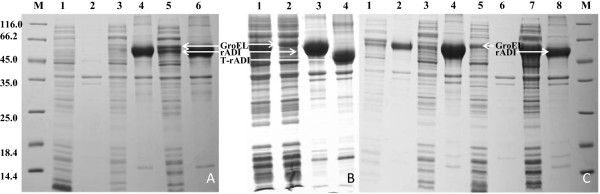
**SDS-PAGE analysis of the production of rADI. (A)** Co-expression with GroES-GroEL chaperones **(B)** Truncation of the N-terminal 30 amino acids. **(C)** The combined addition of D-glucose and L-arginine in LB medium. All the protein samples were normalized at OD_600_. In **(A)**, M, molecular-mass marker protein; lanes 1–2: supernatant and cell debris of the pET-30a(+)-harboring *E. coli* BL21(DE3) cells; lanes 3–4: supernatant and cell debris of pET30a-ADI-harboring *E. coli* BL21(DE3) alone; lanes 5–6: supernatant and cell debris of pET30a-ADI-harboring *E. coli* BL21(DE3) cells co-expressed with GroES-GroEL. In **(B)**, lanes 1–2: supernatants of cells harboring rADI and truncated rADI; lanes 3–4: cell debris of cells harboring rADI and truncated rADI. In **(C)**, lanes 1–2: supernatant and cell debris of cells harboring pET30a-ADI and pGro7 from cultures containing 0.5% L-arginine; lanes 3–4: supernatant and cell debris of cells harboring pET30a-ADI and pGro7 from cultures containing 0.5% D-glucose; lanes 5–6: supernatant and cell debris of cells harboring pET-30a(+) and pGro7 from cultures containing 0.5% L-arginine and 0.5% D-glucose; lanes 7–8: supernatant and cell debris of cells harboring pET30a-ADI and pGro7 from cultures containing 0.5% L-arginine and 0.5% D-glucose; lane M: molecular-mass marker protein.

The arginine deiminase of *P. putida* had been studied due to its characteristics by purification from its original host [22]. Because of its high specific activity (58.8 U/mg) and *in-vitro* solubility, we selected the enzyme to investigate its potential in industrial conversion from L-arginine to L-citrulline. We optimized the gene sequence according to the codon frequency table of *E. coli* BL21(DE3) using the “codon randomization” strategy [23] and synthesized it. The artificial ADI gene (Figure 1) was constructed into different expression plasmids, including pET-30a(+), pBAD/gIIIA, pColdTF and pGEX-6P-1, for overexpression in *E. coli* BL21(DE3). After a normal optimization process according to the previous report [24], 0.1 mM IPTG and 0.04% L-arabinose incubated at 16°C were used to induce the rADI expression in *E. coli* cells. These expression plasmids, except pET-30a(+), exhibited nearly no solubility and enzymatic activity to hydrolyze L-arginine to L-citrulline. The pET30a-ADI produced overexpressed rADI proteins. However, there was only a weak corresponding band (approximately 53 kDa) in the soluble fractions (Figure 2A, lane 3), which gave the hydrolytic activity of 0.64 ± 0.04 U/ml. Most of the expressed proteins were existed in the form of inactive inclusion bodies (Figure 2A, lane 4).

Wen *et al.*[25] reported that truncation of a non-catalytic region in a 1,3-1,4-β-D-glucanase from *Fibrobacter succinogenes* improved enzymatic activity and thermotolerance. The crystal structures of ADI from *Mycoplasma arginini* and *Pseudomonas aeruginosa* showed that the enzymes shared the same catalytic triad (Cys-His-Glu) and the Cys active site [26,27]. The catalytic region mainly located in the C-terminal region of ADI. To explore the effects of deleting the N-terminal non-catalytic fragment on solubility and activity of rADI, our construction based on pET30a-ADI, a plasmid that expressed a truncated ADI protein lacking the N-terminal 30 amino acids (T-rADI, see Figure 1). Unfortunately, instead of improving solubility of the product, the truncation resulted in complete inactivation of the enzyme (Figure 2B, lanes 2 & 4).

### Co-expression with chaperone teams

It is well known that chaperone proteins, such as DnaK-DnaJ-GrpE and GroES-GroEL are efficient to assist protein folding. Thus, improve the production of active recombinant proteins [1,6,28,29]. We co-expressed the pET30a-ADI with four plasmids encoding different chaperone teams respectively. While pGro7 (expressing GroES-GroEL) and pG-KJE8 (expressing DnaK-DnaJ-GrpE and GroES-GroEL) had a similar effect to improve solubility of the rADI proteins, pKJE7 (expressing DnaK-DnaJ-GrpE) and pTf16 (expressing Tig) didn’t play any significant role to improve the solubility (Table 1). It suggested that only the GroES-GroEL chaperone team contributed to the improvement of soluble rADI proteins. For example, when the pET30a-ADI was co-expressed with pGro7, more rADI proteins formed as compared to the single pET30a-ADI plasmid that were present in the soluble part (Figure 2A, lane 5). The total activity was accordingly improved to 3.22 ± 0.06 U/ml, approximately 5-fold higher than that of the rADI expressed alone. However, the rADI proteins were still mostly in inclusion body forms (Figure 2A, lane 6). The further optimizations were performed based on the co-expression with pGro7.

**Table 1:** The total activity of rADI co-expressed with different molecular chaperone

**Plasmid**	**pET30a-ADI**	**+ pG-KJE8**	**+ pGro7**	**+ pKJE7**	**+ pTf16**
Total activity (U/ml)	0.64 ± 0.04	3.14 ± 0.15	3.22 ± 0.06	0.60 ± 0.08	0.74 ± 0.01

### Cultivation-improved solubilization of rADI

Kozai *et al*. [30] reported that the addition of L-arginine in the refolding solution was able to clearly increase the enzymatic activity of denatured ADI proteins. However, our experiments showed that the presence of L-arginine had no effect on the *in-vitro* solubilization of rADI proteins in the inclusion body form. We further tested whether L-arginine is helpful to improve the *in vivo* solubilization of ADI proteins in *E. coli* cells before the formation of inclusion bodies. *E. coli* cells are routinely cultivated in the LB medium, which contains peptones and yeast extracts as the carbon and nitrogen sources. We added different concentrations of L-arginine in the LB medium to cultivate the pET30a-ADI plasmid-harboring *E. coli* BL21(DE3) cells. Low concentrations of L-arginine (less than 1%) had no effect on the solubilization of rADI proteins, while higher concentrations (1%-5%) caused alkalization of the medium (pH10.0), in which *E. coli* cells developed poorly or no growth at all. If we adjusted the media containing high L-arginine concentrations to pH 7.0 or added L-arginine hydrochloride to replace L-arginine, which did not change the pH of the medium significantly, *E. coli* cells were able to grow normally but the solubilization of rADI proteins did not improve.

The bacteria, such as *Streptococcus lactis* and *Oenococcus oeni*, that are able to utilize L-arginine as energy sources were unable to take up L-arginine in the absence of fermentable sugars [31-34]. We suggested that less amount of fermentable sugars in the LB medium might cause no effect of the addition of L-arginine on the solubilization of rADI proteins. Thus, we supplemented D-glucose together with L-arginine in the LB medium. Interestingly, the expression of soluble ADI proteins varied significantly with different concentrations of the supplements. The combination of 0.5% L-arginine and 0.5% D-glucose gave the highest solubility of the protein (Figure 2C), the enzymatic activity of the crude extracts reached 9.31 ± 0.01 to 9.43 ± 0.02 U/ml, which were much higher than that in the other combinations (Table 2). Under this condition, the yield of total and soluble rADI proteins were measured with the Bio-Rad’ Image lab software, increased approximately 1.47 and 7.34-fold higher than that of the alone-expressed rADI and 1.28 and 2.87-fold higher than that of the rADI co-expressed with pGro7 respectively. The total activity increased approximately 15-fold higher than the rADI expressed alone (0.64 ± 0.04 U/ml) and 3-fold higher than that of the co-expression with pGro7 (3.16 ± 0.02 U/ml). In the absence of pGro7, the combination of 0.5% L-arginine and 0.5% D-glucose also enhanced the total activity to 2.37 ± 0.03 U/ml.

**Table 2:** Effects of combined additions of different sugars and amino acids on the enzymatic activities of rADI condition optimization

**Combined addition**^**a**^				**Enzymatic activity**^**b**^**(U/ml)**
**Amino acid**	**Percentage**	**Sugar**	**Percentage**	
			0%	2.08 ± 0.05
			0.5%	9.43 ± 0.02
L-arginine 0.5%		D-glucose	1%	1.76 ± 0.01
			2%	1.60 ± 0.06
			5%	1.41 ± 0.01
	0%			2.78 ± 0.02
	0.5%			9.33 ± 0.04
L-arginine	1%	D-glucose 0.5%		0.86 ± 0.04
	2%			0.74 ± 0.03
	5%			0.70 ± 0.05
	0%			2.92 ± 0.03
	0.5%			2.93 ± 0.05
L-arginine hydrochloride	1%	D-glucose 0.5%		3.85 ± 0.07
	2%			4.35 ± 0.02
	5%			3.95 ± 0.04
	0%			3.18 ± 0.03
	0.5%			3.20 ± 0.08
L-arginine hydrochloride	1%	**-**		3.31 ± 0.05
	2%			3.60 ± 0.02
	5%			3.20 ± 0.08
		**-**	**-**	3.26 ± 0.07
		D-glucose	0.5%	9.31 ± 0.01
L-arginine 0.5%		D-galactose	0.5%	2.35 ± 0.04
		D-maltose	0.5%	1.96 ± 0.04
		D-sucrose	0.5%	2.49 ± 0.10
**-**	**-**			3.22 ± 0.06
L-arginine	0.5%			9.39 ± 0.03
L-tryptophan	0.5%	D-glucose 0.5%		1.93 ± 0.06
L-citrulline	0.5%			1.97 ± 0.01
L-glycine	0.5%			2.69 ± 0.02

^a^: with the presence of pGro7; ^b^: produced from crude extracts.

It was noted that higher concentrations of D-glucose (1% or more) supported better growth of cells, but the activities of crude extracts were prominently decreased. Higher concentrations of supplemented L-arginine (1% or more) inhibited the growth of cells because of alkalization of the medium, even with the presence of D-glucose, which caused no effect in the improvement of enzymatic activities (Table 2). For example, at the concentration of 1% L-arginine (combined with 0.5% D-glucose), the OD_600_ of culture broth was less than 1.0 after 24 hrs of incubation and at the 2% concentration, there were no growth. Surprisingly, when we added different concentrations of L-arginine hydrochloride, instead of L-arginine in the LB medium with D-glucose, no improvement was observed for the solubilization of rADI proteins (Table 2). When L-arginine was combined with other sugars, such as D-galactose, D-sucrose and D-maltose, the solubilization of rADI proteins was not improved. When L-arginine was replaced with non-substrate amino acids of ADI, such as L-tryptophan, L-citrulline and L-glycine no improvement was observed for the solubilization of rADI proteins (Table 2).

We also assayed the effects of the combined addition of 0.5% L-arginine and 0.5% D-glucose on the expression of another ADI, which was from *Listeria welshimeri* serovar 6b str. SLCC5334 (NC_008555). The total activity of the enzyme was 0.1187 ± 0.04 U/ml in the presence of 0.5% L-arginine and 0.5% D-glucose, which was 7.2-fold higher than it expressed alone (0.0165 ± 0.01 U/ml) and 2.9-fold higher than it co-expressed with pGro7 (0.0412 ± 0.02 U/ml). The increase rate was similar to that for the rADI from *Pseudomonas putida*. These results suggested that the combined addition of L-arginine and D-glucose was applicable in solubilization of different ADI proteins.

### Enzymatic and conversion characteristics of rADI

**Figure 3:**
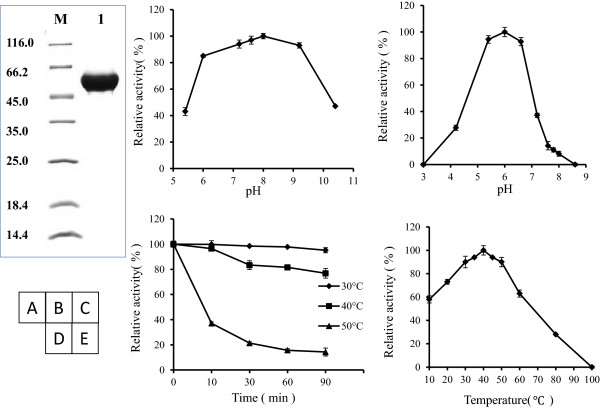
**Enzymatic characteristics of the purified rADI protein. (A)** SDS-PAGE analysis of the purified rADI proteins. The proteins were prepared by co-expression with pGro7 and under the conditions with the addition of 0.5% L-arginine and 0.5% D-glucose. Lanes: M, molecular-mass marker protein; 1, rADI purified using Ni-NTA affinity chromatography. **(B)** The pH stability of the rADI after 12 hrs incubation at 4°C. **(C)** The optimal pH, assayed in buffers ranging from 3.0 to 8.6 at 40°C (Citric Acid-sodium citrate buffer for pH 3.0-5.4, Sodium phosphate buffer for pH 6.0-8.6). **(D)** Thermostability at 30°C (diamond), 40°C (square) and 50°C (triangle). **(E)** The optimal temperature, measured at various points from 10°C to 100°C in 500 mM Sodium phosphate buffer (pH 6.0).

**Figure 4:**
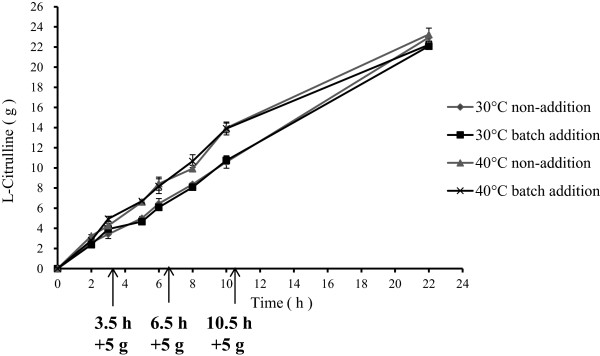
**Conversation curves of L-arginine to L-citrulline by crude rADI enzymes.** 20 g of L-arginine were added at the beginning (-▲- and -■-) or batch-supplemented (-×- and - × -) from 5 g to 20 g at different time points (indicated by black arrows). The conversation experiments were performed at 30°C and 40°C.

After purification using Ni-NTA affinity chromatography, the recombinant proteins showed as a single band with a size of 53 kDa on SDS-PAGE gel (Figure 3A). We assayed enzymatic characteristics of the purified rADI proteins. The rADI, which contained some additional terminal fragments such as a His-tag and some restriction sites, showed similar characteristics as the original ADI obtained from *Pseudomonas putida*[22]. For example, the stability of proteins was observed between pH 7.2 and pH 9.0 after 12 hrs incubation (Figure 3B). The optimal pH was 6.0 for enzymatic activity, but the activity was sharply decreased with the variation of pH value (above 7.2 or below 4.5) (Figure 3C). The enzymatic activity of rADI was mostly retained after incubation at 30°C or 40°C for 90 min, clearly decreased after incubation at 50°C for 90 min (Figure 3D). Cu^2+^ (10 mM), SDS (1 mM, 10 mM) and DTT (1 mM, 10 mM) strongly inhibited the activity of rADI, Cr^3+^ (10 mM), Co^2+^ (10 mM) had a slight inhibition for rADI, while other metal ions had no remarkable effect observed on rADI (Table 3). Interestingly, the suitable temperature for the enzymatic activity of rADI was between 30°C and 50°C and the optimal temperature was 40°C (Figure 3E). The optimal temperature was 50°C for the original ADI measured in *Pseudomonas putida*[22]. At the optimal 40°C, the specific activity of rADI reached 76 ± 0.03 U/mg, which was also higher than 58.8 U/mg of the original at 50°C.We performed conversion experiments using the sodium phosphate buffer (500 mM, pH 6.0) containing 10% L-arginine. The L-citrulline products increased linearly at 30°C or 40°C. After 22 hrs of incubation, 20 g of L-arginine substrates were almost converted into L-citrulline by the crude enzyme from 25 ml cultures. The addition of the batch substrate or not, had no impact on the conversion (Figure 4). The above enzymatic characteristics suggested that rADI was a potential candidate for industrial conversion of L-arginine to L-citrulline.

**Table 3:** The effect of metal ions and reagents on the activity of rADI

**Metal ions/Reagents**	**Relative remaining activity (%) of rADI**	
	**1 mM**	**10 mM**
Control	100.0 ± 2.9	100.0 ± 2.4
Li^+^	99.6 ± 2.5	100.5 ± 0.8
Na^+^	100.1 ± 3.5	102.5 ± 4.4
K^+^	104.1 ± 2.5	100.1 ± 4.9
Mg^2+^	96.4 ± 3.2	102.1 ± 1.5
Ca^2+^	96.2 ± 3.9	91.2 ± 2.4
Mn^2+^	97.4 ± 2.1	101.5 ± 4.2
Cr^3+^	98.1 ± 2.3	81.5 ± 1.9
Co^2+^	94.9 ± 2.8	73.7 ± 2.5
Ni^2+^	100.6 ± 1.9	90.1 ± 0.1
Cu^2+^	80.2 ± 1.2	33.6 ± 1.5
EDTA	100.3 ± 4.6	94.9 ± 1.8
SDS	22.2 ± 2.5	4.9 ± 1.6
DTT	71.0 ± 0.3	13.6 ± 1.2

## Discussion

Previously, it had been observed that the improvement of the substrate L-arginine for correct refolding of denatured ADI proteins in the refolding solution [30]. However, L-arginine had no effect on the *in vitro* solubilization of rADI proteins in inclusion body forms. Thus, *in vivo* solubilization is important for potential industrial applications of rADI proteins. In the laboratories, the *E. coli* cells are routinely cultivated in LB medium with tryptone and yeast extracts as the carbon and nitrogen sources. The present study showed that the combined addition of L-arginine and D-glucose in the LB medium significantly improved *in vivo* solubilization of rADI proteins, but the addition of L-arginine or D-glucose alone did not have the effect. L-arginine have to enter *E. coli* cells to play the *in vivo* solubilization functions. We suggested that the functions of L-arginine in solubilization of ADI were probably via the binding to the proteins, thus preventing the formation of inclusion bodies.

The role of D-glucose is still unknown during this process. There are three possibilities of its roles: (1) D-glucose helped L-arginine to transport through the cell membrane (2) D-glucose balanced the medium pH (3) D-glucose maintained the intracellular pH or metabolic processes. High concentrations of L-arginine caused alkalization of medium, which extensively inhibited the growth of cells. However, L-arginine hydrochloride did not change the medium pH significantly. The addition of the compound had no effect on the solubilization of the rADI proteins, even at low concentrations along with D-glucose. Thus, balancing medium pH or intracellular pH is not the function of D-glucose in the *in vivo* solubilization of rADI proteins.

*Streptococcus lactis* and *Oenococcus oeni* are able to utilize L-arginine as a source of energy via the L-arginine catabolism pathway [33,34]. Poolman *et al.*[33] reported that L-arginine entered these cells by an arginine-ornithine antiporter in *S. lactis*, which was initiated in the presence of a fermentable sugar. Only after initiated, L-arginine was able to subsequently enter cells via the ornithine and arginine concentration gradients formed during arginine metabolism [31,32]. It was suggested fermentable sugars provided the energy for the entrance of L-arginine into cells. Bioinformatics analysis showed that the genome of *E. coli* BL21(DE3) (GenBank accession no. CP001509) had some predicted proteins related to the arginine-ornithine antiporter, such as an arginine/ornithine antiporter transporter, an arginine/ornithine transporter subunit and an arginine/ornithine transport system ATPase [35]. We supposed that the increased solubilization was associated with the *in vivo* interactions between L-arginine and newly synthesized rADI proteins during the process of refolding, which requires further determination.

## Conclusions

Combined addition of L-arginine and D-glucose in the LB medium significantly improved *in vivo* solubility of rADI proteins but the addition of L-arginine or D-glucose alone did not have the effect. With the support of GroEL-GroES, the promotion of combined D-glucose and L-arginine was more significant. The studies present in this paper suggested a new strategy to increase the solubilization of overexpressed recombinant proteins in *E. coli* cells.

## Methods

### Strains and cultivation conditions

*E. coli* DH5α and BL21(DE3) strains (Table 4) were used as the hosts for cloning and expression of the ADI gene, respectively. The *E. coli* cells were routinely cultivated in Luria-Bertani (LB) medium (containing tryptone 10 g/L, yeast extract 5 g/L and NaCl 10 g/L).

**Table 4:** Bacterial strains and plasmids used in this study

**Strains/plasmids**	**Genotype or description**	**Source**
Strains		
*E. coli* DH5α	F^-^,*sup*E44,*Δlac*U169(φ*80lacZΔ*M15),*hsd*R17, *rec*A1,*end*A1, *gyr*A96, *thi*-1, *rel*A1	Life Technologies
*E. coli* BL21(DE3)	F^-^, *omp*T, *hsd*S_B_(r_B_-m_B_-), *gal, dcm*(DE3)	Life Technologies
Plasmids		
pG-KJE8	Encoding DnaK-DnaJ-GrpE / GroES-GroEL	Takara
pGro7	Encoding GroES-GroEL	Takara
pKJE7	Encoding DnaK-DnaJ-GrpE	Takara
pTf16	Encoding Tig	Takara
pColdTF	Expression vector	Takara
pET-30a (+)	Expression vector	Novagen
pGEX-6P-1	Expression vector	GE Healthcare
pBAD/gIIIA	Expression vector	Invitrogen

### Construction of recombinant plasmids

The ADI gene studied in this study is taken from *P. putida* (GenBank accession no. P41142). We optimized the gene sequence according to the codon frequency table of *E. coli* BL21(DE3) using the “codon randomization” strategy [23]. The artificial gene and its corresponding amino acid sequences (Figure 1) were deposited in GenBank under the accession no. KJ411883. After synthesized at Biosune (Shanghai, China), the gene was amplified using a specific primer pair (Forward: CATG*CCATGG*CTATGAGCGCCGAAAAACAGAAGTATG; Reverse: CCC*AAGCTT*GATAATCGATAGGGTCGCGAACAATG) to generate the digestion sites of endonucleases *Nco*I and *Hin*dIII (italics). After purification, the amplified fragment was digested using the restriction enzymes and ligated into plasmid pET-30a(+), named pET30a-ADI, which was confirmed by sequencing. The construction of recombinant plasmids, transformation and protein expression were performed according to the protocol described by Sambrook and Russell [36].

### Co-expression with the chaperone teams

The plasmids pGro7, pG-KJE8, pTf16 and pKJE7 encode different chaperone teams (Table 4). The pET30a-ADI was co-expressed with these four plasmids respectively. *E. coli* BL21(DE3) cells harboring plasmid pET30a-ADI alone or together with a chaperone-encoding plasmid were cultivated in 5 ml of LB medium containing 40 μg/ml kanamycin and 20 μg/ml chloramphenicol for the selection of recombinants. After overnight shaking at 37°C, the culture was inoculated (1%) into fresh LB medium containing antibiotics and 0.04% L-arabinose (pG-KJE8 was induced by 5 μg/ml tetracycline and 0.04% L-arabinose). The culture was shaken at 37°C to induce the expression of chaperones. When the OD_600_ of the culture reached 0.6-0.8, isopropyl-β-D-thiogalactopyranoside (IPTG) was added to a final concentration of 0.1 mM to induce the expression of rADI. After additional 24 hrs of incubation at 16°C, the cells were harvested by centrifugation at 8,000 × *g* and 4°C for 10 min. The cell pellets were washed, resuspended in Tris-NaCl buffer (50 mM Tris, 150 mM NaCl, pH 8.0) and broken by sonication on ice. After centrifugation at 12,000 × *g* and 4°C for 30 min, the separated supernatant and cell debris were assayed enzymatic activities and in sodium dodecyl sulfate-polyacrylamide gel electrophoresis (SDS-PAGE, 10% gel concentration).

### Cultivation-improved solubilization of rADI

The *E. coli* BL21(DE3) cells harboring the combined plasmids of pET30a-ADI and pGro7 were cultivated as described above. To assay the effects of cultivation on the solubility of rADI, different saccharides (D-glucose, D-sucrose, D-galactose or D-maltose) were added to a final concentration of 0.5% in the LB medium containing the antibiotics and L-arabinose. Together with the addition of IPTG, different amino acids (L-arginine, L-tryptophan, L-citrulline and L-glycine) were added to a final concentration of 0.5% when the culture OD_600_ reached 0.6-0.8. The cultures were incubated for additional 24 hrs at 16°C. *E. coli* BL21(DE3) harboring pET-30a(+) was used as negative control.

### Purification of rADI

After harvested, cells were broken by sonication, and the supernatants were applied to the Ni-NTA affinity chromatography (GE Healthcare, Buckinghamshire, U.K.) to purify rADI. The protein concentration was determined using the Bradford method (Bio-Rad, Munich, Germany). Different fractions were collected and assayed enzymatic activities and in SDS-PAGE (10%).

### Activity assays of rADI

The rADI activity was determined using a modified method of diacetyl monoxime thiosemicarbazide [17,37]. 40 mM L-arginine was dissolved in 500 mM sodium phosphate buffer (pH 6.0). After the addition of rADI, the mixture was incubated at 40°C for 10 min. Then, acid-ferric solution (containing 15% H_2_SO_4_, 60% H_3_PO_4_ and 0.005% FeCl_3_) and DAM-TSC solution (containing 1% diacetyl monoxime and 0.06% thiosemicarbazide) were added to stop the reaction and the mixture was heated at 100°C for 10 min for coloration. The amount of L-citrulline was evaluated by measuring the optical density at 530 nm. The protein concentration was determined using Bradford-assay with bovine serum albumin as standard. One unit of the ADI activity was defined as the amount of enzymes to produce one μmol L-citrulline per minute under the assay conditions. All the assays were performed in triplicate. The total enzymatic activity was defined as units per milliliter of culture and the specific enzymatic activity was shown as units per milligram of purified enzyme.

### Conversion rate of crude enzyme

The crude enzymes were prepared from sonication-broken cells using the above-described methods. 20 ml crude extracts from 100 ml culture broth were divided into four equal parts. The conversion of L-arginine to L-citrulline was assayed in 200 ml reaction buffer (500 mM sodium phosphate buffer, pH 6.0) at 30°C or 40°C. 20 g L-arginine was added at the beginning or batch-supplemented from 5 g to 20 g at different time points.

## Competing interests

The authors declare that they have no competing interests.

## Authors’ contributions

YZL and YW designed the experiments and analyzed the data. YW performed all the experiments. YZL and YW wrote the paper. Both authors read and approved the final manuscript.
